# Regionally restricted modulation of *Sam68* expression and *Arhgef9* alternative splicing in the hippocampus of a murine model of multiple sclerosis

**DOI:** 10.3389/fnmol.2022.1073627

**Published:** 2023-01-12

**Authors:** Annalisa Adinolfi, Gabriele Di Sante, Luca Rivignani Vaccari, Maria Tredicine, Francesco Ria, Davide Bonvissuto, Valentina Corvino, Claudio Sette, Maria Concetta Geloso

**Affiliations:** ^1^Section of Human Anatomy, Department of Neuroscience, Università Cattolica del Sacro Cuore, Rome, Italy; ^2^Section of Human, Clinic and Forensic Anatomy, Department of Medicine and Surgery, University of Perugia, Perugia, Italy; ^3^Section of General Pathology, Department of Translational Medicine and Surgery, Università Cattolica del Sacro Cuore, Rome, Italy; ^4^GSTEP-Organoids Core Facility, Fondazione Policlinico Agostino Gemelli IRCCS, Rome, Italy

**Keywords:** multiple sclerosis, experimental autoimmune encephalomyelitis, parvalbumin, neuroinflammation, alternative splicing, hippocampus, *Arhgef9*, *Sam68*

## Abstract

Multiple sclerosis (MS) and its preclinical models are characterized by marked changes in neuroplasticity, including excitatory/inhibitory imbalance and synaptic dysfunction that are believed to underlie the progressive cognitive impairment (CI), which represents a significant clinical hallmark of the disease. In this study, we investigated several parameters of neuroplasticity in the hippocampus of the experimental autoimmune encephalomyelitis (EAE) SJL/J mouse model, characterized by rostral inflammatory and demyelinating lesions similar to Relapsing–Remitting MS. By combining morphological and molecular analyses, we found that the hippocampus undergoes extensive inflammation in EAE-mice, more pronounced in the CA3 and dentate gyrus (DG) subfields than in the CA1, associated with changes in GABAergic circuitry, as indicated by the increased expression of the interneuron marker Parvalbumin selectively in CA3. By laser-microdissection, we investigated the impact of EAE on the alternative splicing of *Arhgef9*, a gene encoding a post-synaptic protein playing an essential role in GABAergic synapses and whose mutations have been related to CI and epilepsy. Our results indicate that EAE induces a specific increase in inclusion of the alternative exon 11a only in the CA3 and DG subfields, in line with the higher local levels of inflammation. Consistently, we found a region-specific downregulation of Sam68, a splicing-factor that represses this splicing event. Collectively, our findings confirm a regionalized distribution of inflammation in the hippocampus of EAE-mice. Moreover, since neuronal circuit rearrangement and dynamic remodeling of structural components of the synapse are key processes that contribute to neuroplasticity, our study suggests potential new molecular players involved in EAE-induced hippocampal dysfunction.

## 1. Introduction

Mounting evidence indicates the concomitant occurrence of primitive neurodegeneration of grey matter (GM) structures in Multiple Sclerosis (MS), independent from demyelination (Calabrese et al., 2015). Grey matter damage is believed to provide a substantial contribution to the progressive impairment of cognitive functions (Mandolesi et al., 2015) that is observed in many MS patients and is now recognized as a significant clinical hallmark of the disease (Chiaravalloti et al., 2015). While the pathogenic mechanisms underlying such primitive neurodegeneration have not been fully elucidated, early synaptic dysfunction is believed to play an important role in the progressive cognitive impairment (CI) observed in patients (Calabrese et al., 2015; Di Filippo et al., 2018).

Functional properties of the synapses rely on the timely expression of synaptic proteins and the proper structural assembly of synaptic contacts on dendritic spines (Südhof, 2017; Nakahata and Yasuda, 2018; Batool et al., 2019). More recently, a growing body of evidence indicates that the expression of synaptic proteins is also finely tuned by alternative splicing (AS), a molecular process that allows to express multiple protein isoforms from a single gene (Furlanis and Scheiffele, 2018; Naro et al., 2021). Indeed, although AS represents a powerful tool for achieving protein diversification in most eukaryotic cells and tissues (Paronetto et al., 2016; Baralle and Giudice, 2017), this process is particularly relevant in the brain, likely due to its functional and structural complexity (Naro et al., 2021). Comparative transcriptome analyses highlighted the expression of a huge repertoire of splice variants in the central nervous system (CNS; Furlanis and Scheiffele, 2018), where orchestrated splicing programs play instructive roles in the development of neuronal–specific properties, establishment of functional circuits and synapse specification (Raj and Blencowe, 2015; Furlanis and Scheiffele, 2018). Neuron-specific splice variants of the same gene exert different functional roles in neurons. For instance, inclusion of a single exon (named alternatively spliced 4 or AS4) in the Neurexin *(Nrxn)1–3* genes, encoding presynaptic adhesion molecules relevant for cognition, was shown to dictate the selection of post-synaptic receptors (Südhof, 2017). On the other hand, disruption of the splicing pattern of genes encoding neuronal proteins has been associated with several neurological diseases (Dredge et al., 2001). About this topic, recent studies reported an association between GM damage in MS patients and dysfunction of specific splicing regulators (Salapa et al., 2018, 2020; Libner et al., 2020; Masaki et al., 2020). In this regard, we previously demonstrated that inflammation induces changes in *Nrxn1-3* AS4 splicing in the prefrontal cortex (PFC) of experimental autoimmune encephalomyelitis (EAE) mice, an animal model of MS, and that this regulation was associated with CI in behavioral tasks (Marchese et al., 2021). Moreover, such changes were correlated with inflammation-dependent reduction of *Slm2* (Marchese et al., 2021), one of the members of the Signal Transduction Associated RNA-binding (STAR) family of RNA-binding proteins. STAR proteins include SRC associated in mitosis of 68 kDa (Sam68), Sam68-like mammalian protein (SLM)1 and SLM2, all important regulators of AS of neuronal genes in the CNS (Iijima et al., 2011, 2014; Ehrmann et al., 2013; Farini et al., 2020). SLM1 and SLM2 are exclusively expressed by neurons in a cell-type and region-specific manner. SLM1 is mainly present in glutamatergic dentate granule cells and in a subset of cholecystokinin–calbindin double-positive (+) inhibitory interneurons, while SLM2 predominates in pyramidal neurons and somatostatin+ GABAergic interneurons of the *Cornu Ammonis* (Iijima et al., 2014; Ehrmann et al., 2016). In contrast, Sam68 is ubiquitously expressed by both neurons and glial cells and it was shown to regulate AS of several synaptic genes in both GABAergic and glutamatergic synapses (Iijima et al., 2019; Witte et al., 2019; Farini et al., 2020).

In this study, we have investigated the impact exerted by EAE on AS of synaptic genes in the hippocampus, a brain structure involved in memory and learning that is characterized by a remarkable capacity of structural reorganization and plasticity throughout the lifespan (Leuner and Gould, 2010). We focused on a restricted set of genes regulated by the STAR family members, with the aim to provide insights in the molecular and cellular processes involved in CI in MS and to identify new potential molecular targets for therapy.

## 2. Materials and methods

### 2.1. Immunization and EAE induction

Experimental autoimmune encephalomyelitis was induced in female SJL/J mice (6–8 weeks old; Charles River Laboratories, Calco, Italy) as described (Marchese et al., 2021; Tredicine et al., 2022). Briefly, mice were subcutaneously injected, on day post immunization (d.p.i.) 0 and 7, with an emulsion of 100 μl/mouse containing 75 μg/mouse of PLP_139-151_ (PRIMM, Milan, Italy) and enriched complete Freund’s adjuvant (CFA 8X concentrated, Merck, Milan, Italy), in a final volume of 100 μl/mouse. Intraperitoneal (i.p.) administration of *Bordetella Pertussis* toxin (300 ng/mouse, Merck, Milan, Italy) was performed on d.p.i. 0, 1, 7, and 8 (Penitente et al., 2008; Nicolò et al., 2010; Piermattei et al., 2016; Marchese et al., 2021). Clinical Score and Signs (CSS) of EAE were daily evaluated as previously described (Di Sante et al., 2020; Camponeschi et al., 2021; Marchese et al., 2021). Method details are provided in supplementary information.

### 2.2. Tissue processing

#### 2.2.1. Euthanasia

EAE mice were euthanized at the beginning of the acute phase of the disease (CSS = 2–3; d.p.i 14 ± 2; *n* = 8) and CTRLs (*n* = 3–5) were sacrificed accordingly. Under deep anesthesia (87.5 mg/Kg Ketamine + 12.5 mg/Kg xylazine; 0.1 ml/20 g mouse weight, i.p.), mice were perfused with sterile saline solution, the brain was removed and one hemisphere, after 4% paraformaldehyde (PFA) fixation (48 h), was intended for morphological analysis, while the hippocampus extracted from the other hemisphere was used for molecular assays and processed accordingly.

#### 2.2.2. Molecular biology assays

##### 2.2.2.1. Real time PCR

Total RNA was extracted from hippocampal samples using Trizol protocol (Invitrogen, Carlsbad, CA, United States). Reverse transcription and quantitative real time PCR (qPCR) were used to amplify the selected targets or splice variants using the Power-SYBRgreen PCR master mix (Roche, Mannheim, Germany), sequence-specific primers and the StepOne Real-Time PCR System. Target gene intensities were normalized to the reference housekeeping gene (L34) and quantified as previously described (Corvino et al., 2015; Marchese et al., 2021).

##### 2.2.2.2. Detection of splicing patterns

Total RNA obtained from hippocampal samples was used for RT-PCR analyses performed using GoTaq^®^ G2 (Promega, Italy) and primers that distinguish between splice variants of the selected genes (Supplementary Table S1). Reactions were quantitated by densitometric analyses of agarose or polyacrylamide gels (Marchese et al., 2021). Splicing profiles were calculated as percentage of splicing inclusion (PSI), which refers to the percentage of transcripts including the variable exon (Ehrmann et al., 2013).

#### 2.2.3. Immunohistochemistry

After fixation and cryoprotection in sucrose 30%, 30 μm thick serial coronal hippocampal sections were cut with a freezing microtome, taken from 1,34 to 2,18 bregma coordinates (Paxinos and Franklin, 2013). Every sixth section was stained with Nissl-staining for histologic analysis or processed by immunocytochemistry to detect the microglial marker Iba1 (Wako, Richmond, VA, United States; 1:1,000), the marker of activated microglia CD68 (Immunological Sciences, Rome, Italy; 1:200) and the interneuron marker Parvalbumin (PV; NOVUS Biologicals, Centennial, CO, United States 1:2,000). Reactions were revealed using the appropriate secondary antibodies (goat anti-rabbit-FITC, Vector, Burlingame, CA, United States, 1:200 or donkey anti-mouse Cy3, Jackson Immunoresearch Laboratories, West Grove, PA, United States, 1:400) and counterstained with Neuro-Trace (Thermo Fisher Scientific, Waltham, MA, United States; Marchese et al., 2018, 2021).

Confocal laser scanning microscope (LSM510 META, Zeiss, Oberkochen, Germany) was employed to analyze the colocalization of the markers investigated.

## 3. Quantitative analysis

### 3.1. Neuropathological evaluation

As previously described (Marchese et al., 2021), in all EAE-animals we analyzed the numbers of infiltrates/mm^2^ and the percentage of area infiltrated by mononuclear cells in the following brain areas: cerebral cortex, striatum, thalamus, hippocampus, white matter (corpus callosum, internal capsule, and fimbria). Method details are provided in supplementary information.

### 3.2. Unbiased stereology

The optical fractionator stereological design was used to obtain unbiased counts of total numbers of PV + interneurons in CA1, CA3 and dentate gyrus (DG), using the Stereo Investigator system (Stereo Investigator software, Version 9, MicroBrightField Europe, Germany), essentially as previously described (Corvino et al., 2015; Marchese et al., 2018).

Method details are provided in supplementary information.

### 3.3. Confocal microscope quantitative analysis of double-stained cells

Iba1/CD68 double-stained cells were quantified in the different hippocampal subfields of mice from the different experimental groups using z-scan confocal microscopy at 40 × magnification, as previously described (Corvino et al., 2015; Marchese et al., 2018, 2021). The quantification of double-stained cells was expressed as the percentage of Iba1/CD68-double-labeled cells in relation to the total number of Iba1+ cells (Corvino et al., 2015; Marchese et al., 2018, 2021).

### 3.4. Microglial cell density

Semiquantitative analysis of Iba1 + microglia cell density (Iba1 + cells/mm^2^) was performed in CA1, CA3, and DG hippocampal regions of mice from all experimental groups, as described by others (Zhao et al., 2018). Z-stack confocal microglia images were acquired at 40X magnification in 1-in-12 series of sections, and the number of Iba1+ cells/mm^2^ was counted using the Java ImageJ image processing and analysis program (1997–2018, NIH). Each image was opened in ImageJ, keeping a constant thresholding between each image, and converted to a maximum intensity Z-stack projection. Iba1+ cells were then manually counted in specific region of interest to evaluate cell density.

## 4. Laser microdissection technology

As previously described (Farini et al., 2020), 10 μm frozen sections were cut on a cryostat (Leica CM1850) and mounted on PET-membrane 1.4 μm frame slides (Leica) previously cleaned with RNase away (Molecular Bio Products) and UV-treated for 45 min under sterile hood. Modified Cresyl Violet staining for RNA research [0.5 g Cresyl Violet into 50 ml 100% ethanol (VWR, Milano, Italy)] was performed to visualize the hippocampus. Granule cell layer of DG and pyramidal cell layer of CA1 and CA3, in which glial contamination is poorly relevant, were microdissected with a laser-microdissection system (Leica LMD6) and collected in RNA later reagent (QIAGEN, Helden, Germany). Total RNA was extracted from the dissected specimen using a RNAeasy Micro Kit (QIAGEN, Helden, Germany) and quantified with Agilent Bioanalyzer 2,100 using RNA6000 picoKit, according to the manufacture instructions. cDNA was reverse transcribed using SuperScript-IV VILO master Mix with EZ Dnase (Invitrogen, Massachusetts, United States).

## 5. Statistical analysis

Student’s *t*-test, Repeated Measure (RM) ANOVA with EAE as between factor and hippocampal regions as within factor were performed as previously described (Geloso et al., 1998; Corvino et al., 2015) to examine the effects and possible interactions of independent variables (Stat View or GraphPad 6.0 software). When appropriate, *post hoc* comparisons were made using Tukey’s HSD, with a significance level of *p* < 0.05. Pearson’s correlation was performed to explore relationship between two variables.

## 6. Results

### 6.1. Experimental autoimmune encephalomyelitis induces neuroinflammatory changes in the hippocampus of SJL/J mice in the acute phase of the disease

All immunized mice developed clinical signs typical of EAE starting from the 12th–14th d.p.i. and were sacrificed at the beginning of the acute phase (CSS = 2–3; Supplementary Figure S1A). Neuropathological evaluation of Nissl-stained brain sections revealed the presence of numerous subpial and perivascular inflammatory infiltrates, scattered in white matter structures, as well as in different cortical areas, including the hippocampus, and in subcortical grey nuclei (Supplementary Figures S1B–D).

**Figure 1:**
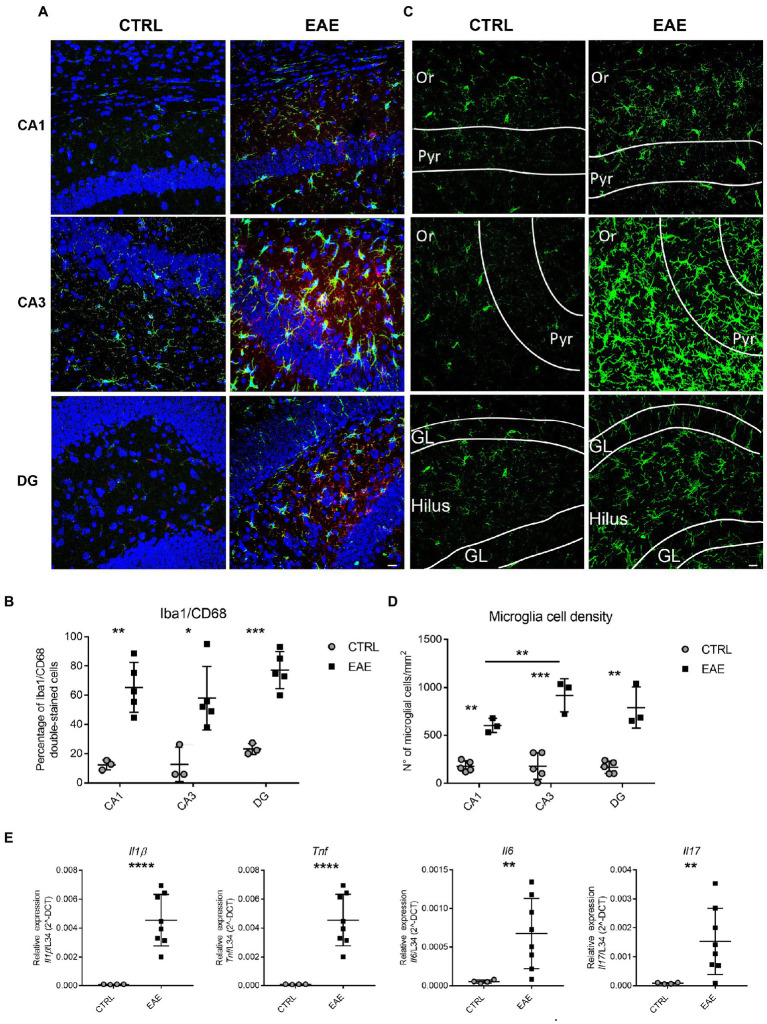
Experimental autoimmune encephalomyelitis (EAE) induces inflammatory changes in the hippocampus of EAE SJL/J mice at the beginning of the acute phase. **(A)** Representative confocal microscopy images of CA1, CA3 and DG hippocampal regions immunostained for the microglial marker Iba1 (green) and the microglial activation marker CD68 (red) and counterstained with Neurotrace (blue) from CTRL and EAE mice. EAE-animals show more numerous double-labelled Iba1/CD68 cells compared with CTRLs. Scale bar: 10 μm. **(B)** Quantitative analysis shows a significantly higher percentage of Iba1/CD68 double-stained cells in all hippocampal regions of EAE animals compared with CTRLs (RM ANOVA: EAE factor: F_1-12_ = 33.12, *p* = 0.0012; hippocampal region: F_2-12_ = 3.9, *p* = 0.049; interaction: F_2-12_ = 0.364, *p* = 0.7); Student’s t test: CA1 ^**^p = 0.0021, CA3 ^*^p = 0.017, DG ^***^p = 0.0004). **(C)** Representative micrographs of Iba1+ hippocampal sections from CTRL and EAE mice pointing out an increase of FIGURE 1 (Continued)Iba1-immunoreactivity in all hippocampal subfields of EAE-mice compared to CTRLs and highlighting a prevalent distribution in CA3. (Or: stratum oriens; Pyr: pyramidal cell layer; GL: granular cell layer). **(D)** Quantitative analysis of Iba1+ cell density in the CA1, CA3 and DG hippocampal regions shows a significant increase of microglial cell density in all hippocampal subfields of EAE mice compared with CTRLs. Notably, a higher microglial cell density is detectable in the CA3 region compared to CA1. [RM-ANOVA: EAE factor: F_1–12_ = 99.1, *p* < 0.0001; hippocampal regions: F_2–12_ = 3.89, *p* = 0.048; interaction: EAE* hippocampal regions: (F_2–12_ = 3.89, *p* = 0.049); Tukey *post hoc* test: CA1 (^**^p = 0.0054), CA3 (^***^p = 0.00017) and DG (^**^p = 0.0031)] (CTRL: *n* = 5, EAE: *n* = 3). **(E)** qPCR analysis performed on total mRNA extracted from the hippocampi of both experimental groups showing a significant increase of *Il1β*, *Tnfα* (Student’s *t*-test ^****^p < 0.0001), *Il6* (Student’s *t*-test, ^**^p = 0.01) and *Il17* (Student’s *t*-test ^**^p = 0.01) expression in EAE-animals compared with CTRLs (*n*: CTRL = 4, EAE = 8). All values are given as mean ± SD.

To assess the inflammatory status of the hippocampus of EAE mice, we then evaluated microglia activation by immunofluorescence. Double-staining with the microglial marker Iba1 and the activation marker CD68 (Slepko and Levi, 1996) showed a significant activation of microglia in all hippocampal subfields of EAE mice compared to CTRLs (Figures 1A,B). Since activated microglia undergo proliferation, we also carried out semi-quantitative evaluation of microglia cell-density in different hippocampal subfields. This analysis highlighted a significant increase of Iba1 + cells in all hippocampal subregions of EAE mice compared with CTRLs (Figures 1C,D). In addition, a significantly enhanced density of Iba1+ cells was detectable in the CA3 region compared to CA1 (*p* = 0.039; Figure 1D). Consistently, the CA3 subfield of EAE mice showed a higher fold change (FC) increase in microglial cell-density (FC = 4.1) than DG (FC = 3.75) and CA1 (FC = 2.3; Figures 1C,D). In addition, hippocampal microglia exhibited the typical phenotypic features of activation, such as larger cell bodies and thicker processes (Hammond et al., 2020), compared with those detectable in the CTRL group. These features were particularly evident in the CA3 subfield and in the DG (Figure 1C). Lastly, qPCR analyses showed a significant increase in the expression of the pro-inflammatory cytokines *Interleukin (Il) 1β*, *Tumor necrosis factor (Tnf) α* (Student’s *t*-test, *p* < 0.0001), *Il6* (Student’s *t*-test, *p* = 0.016) and *Il17* (Student’s t test *p* = 0.017) in the hippocampus of EAE mice (Figure 1E), thus confirming its inflammatory status at molecular level.

Taken together, these results indicate that EAE induces marked inflammatory changes in the hippocampus of SJL/J mice and suggest that the extent of inflammation is particularly relevant in the CA3 and DG subfields.

### 6.2. Experimental autoimmune encephalomyelitis induces local changes in hippocampal circuitry at the onset of disease

To investigate whether inflammation affects hippocampal circuitry, we focused on PV-expressing GABAergic interneurons, which are known to be susceptible to pathological events, such as oxidative stress (Jiang et al., 2013) and neuroinflammation (Jenkins et al., 2009). Moreover, a marked modulation of PV + interneurons has been reported in cortical regions of both MS patients and EAE mice (Nisticò et al., 2013; Magliozzi et al., 2021; Marchese et al., 2021).

**Figure 2:**
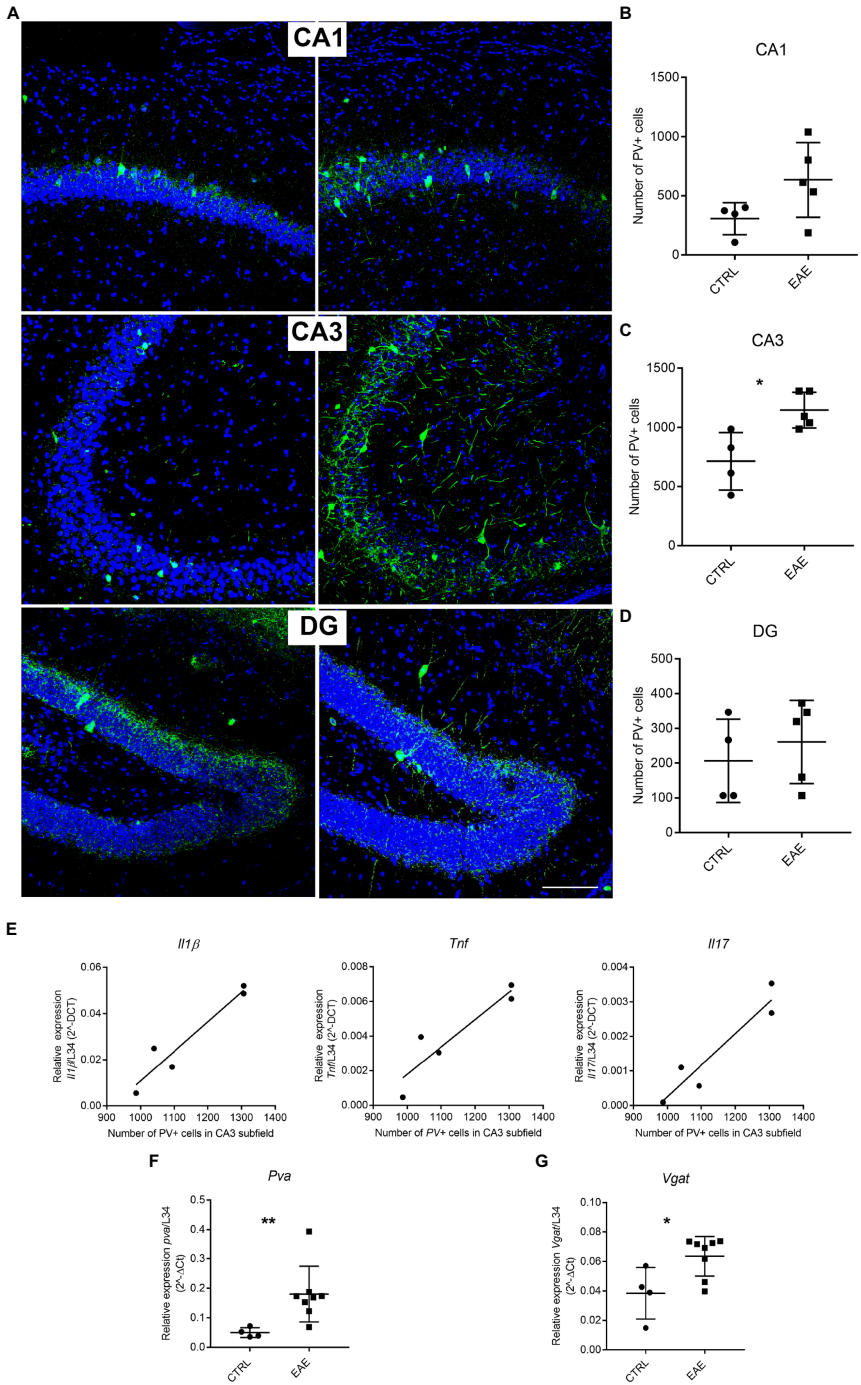
Experimental autoimmune encephalomyelitis (EAE) induces changes in Parvalbumin (PV) expression. **(A)** Representative micrographs of PV-immunostained (green) hippocampal sections counterstained with Neurotrace (blue) from CTRL and EAE mice. Increased PV-immunoreactivity is FIGURE 2 (Continued)detectable in CA1 and CA3 subfields of EAE mice. Scale bar: 150 μm. **(B–D)** Unbiased stereological cell counts confirmed the presence of significantly higher numbers of PV+ cells in the CA3 subfield of EAE-mice compared with CTRLs, while no significant difference emerged in CA1 and DG [RM-ANOVA: EAE factor: F_1–14_ = 11.38, *p* = 0.011, hippocampal regions: (F_2–14_ = 29.6, *p* < 0.0001), EAE*hippocampal regions (F_2–14_ = 2.25, *p* = 0.14). Student’s *t*-test (Tybout et al., 2001): CA3-EAE vs. CA3-CTRL: ^*^p = 0.021; CA1-EAE vs. CA1-CTRL: *p* = 0.09; DG-EAE vs. DG-CTRL: *p* = 0.5 (*n*: CTRL = 4; EAE = 5)]. **(E)** The number of PV+ cells in the CA3 subfield shows a positive correlation with the expression levels of inflammatory cytokines *Il1β*, *Tnfα* and *Il17* (Pearson’s correlation: CA3 PV + cells vs. *Il1β*: *p* = 0.0324, *R* square = 0.9175; CA3 PV + cells vs. *Tnfα*: *p* = 0.0132, *R* square = 0.923; CA3 PV + cells vs. *Il17*: *p* = 0.0151, *R* square = 0.957). **(F,G)** qPCR analysis performed on total mRNA extracted from the hippocampi of EAE and CTRL animals shows a significant increased expression of *Pva* (Student’s *t*-test, ^**^p = 0.0011) and *Vgat* (Student’s *t*-test, ^*^p = 0.0291) genes in EAE animals compared with CTRLs. All values are given as means ± SD.

Immunofluorescence analysis followed by quantitative stereological cell counts pointed out the presence of higher numbers of PV+ cells in the pyramidal layer of the CA3 subfield of EAE mice compared with the CTRL group (Figures 2A–D). A similar trend, albeit not significant, was also observed in the CA1 subfield, whereas no changes were detected in the DG of EAE mice (Figures 2A–D). Notably, the number of PV-expressing interneurons in the CA3 region showed a significant positive correlation with the expression levels of the inflammatory cytokines *Il1β, Tnfα* and *Il17* (Figure 2E). Analysis by qPCR also showed a significant upregulation of the *Pva* gene in the hippocampus of EAE mice compared to the CTRL group (Student’s *t*-test, *p* = 0.0011; Figure 2F), thus confirming the morphological analyses. Consistently, we also detected a significant increase in the expression of the vesicular GABA transporter (*Vgat*), a marker of GABAergic synapses (Chaudhry et al., 1998), in the hippocampus of EAE mice (Student’s *t* test: *p* = 0.0029; Figure 2G).

These results suggest that local inflammation affects GABAergic circuitry in the hippocampus of SJL/J mice, with a prominent involvement of the CA3 subfield.

### 6.3. Region-specific regulation of *Arhgef9* splicing pattern in the hippocampus of EAE mice

**Figure 3:**
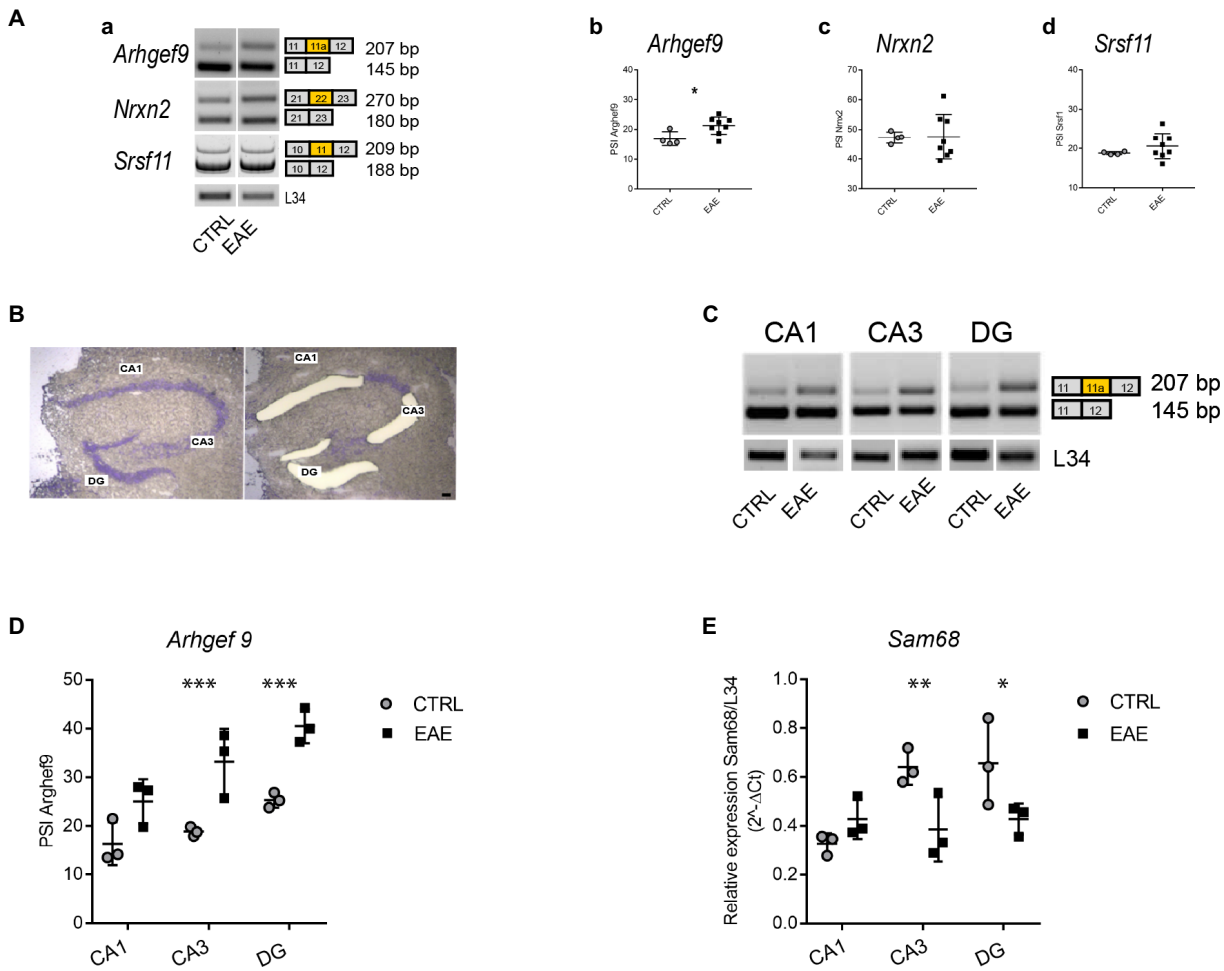
Experimental autoimmune encephalomyelitis (EAE) induces region-specific regulation of the *Arhgef9* splicing pattern and *Sam68* expression. **(A)** Analysis of the splicing pattern of synaptic genes that are target of STAR proteins, performed in the whole hippocampus of CTRL and EAE mice. **(a)** Representative agarose-gel images of the splicing pattern of *Arhgef9* at exon 11a, *Nrxn2* at alternatively spliced segment (AS) 4, and polyacrylamide-gel image of *Srsf11* at exon 11 in CTRL and EAE mice; **(b–d)**: densitometric quantifications of the percentage of splicing inclusion (PSI) at alternatively spliced exon 11a of *Arhgef9* **(b)**, at segment AS4 of *Nrxn2* **(c),** at exon 11 of *Srsf11* **(d).** While no significant modulation of PSI is evident for *Nrxn2* (Student’s *t*-test *p* = 0.94) and *Srsf11* (Student’s *t*-test *p* = 0.3), a significantly increased PSI of the exon 11a of *Arhgef9* is detectable in the EAE group compared to CTRLs (Student’s *t*-test, ^*^p = 0.0279). **(B)** Representative images of the same Nissl-stained hippocampal coronal section before and after laser microdissection of CA1 and CA3 pyramidal cell layer and DG granular cell layer. Scale bar: 150 μm. **(C)** Representative agarose-gel images of the splicing pattern of exon 11a of *Arhgef9* gene in microdissected hippocampal subfields from CTRL and EAE mice. **(D**) Densitometric quantification of PSI of exon 11a of *Arhgef9* in CA1, CA3 and DG hippocampal subfields of CTRL and EAE-mice (RM-ANOVA: EAE factor: F_1,12_ = 21.9; *p* = 0.003, hippocampal regions: F_2,12_ = 25.34; *p* < 0.0001; interaction regions *EAE factor: F_2,12_ = 3.97; *p* = 0.047). Tukey’s *post hoc* multiple comparisons showed a significant increase of the PSI of *Arhgef9* in both CA3 (^***^p = 0.0001), and DG (^***^p = 0.0004) subfields of EAE mice compared with CTRLs, while no significant difference was detected in the CA1 subregion (*p* = 0.1). **(E)** qPCR analysis of *Sam68* expression levels performed on RNA extracted from microdissected hippocampal subfields of EAE and CTRL mice. A significant downregulation restricted to CA3 and DG regions was detectable (RM-ANOVA: hippocampal regions: F_2,8_ = 15.3; *p* = 0.0018; hippocampal regions *EAE: F_2,8_ = 21.8; *p* = 0.006, EAE factor: F_1,8_ = 2.1; *p* = 0.21). Tukey’s *post hoc* test highlighted a significant downregulation of *Sam68* expression levels in both CA3 (^**^p = 0.0035) and DG (^*^p = 0.017) regions of EAE mice compared with CTRLs, while no significant difference was detectable in the CA1 subregion (*p* = 0.1). All values are given as means ± SD.

We then investigated the effects of EAE lesions on the splicing pattern of synaptic genes in the hippocampus, where some splicing factors show a marked regional distribution (Naro et al., 2021). Since we previously reported the involvement of SLM2 in the splicing changes in the PFC of EAE mice (Marchese et al., 2021), we focused on STAR proteins. First, we analyzed the specific synaptic targets of either SLM1, SLM2 or Sam68 on samples of the whole hippocampus taken from EAE and CTRL mice. We selected genes encoding synaptic proteins involved in cognition, such as *Srsf11,* in which the skipping of exon 11 is selectively regulated by SLM1 (Iijima et al., 2019) and has been recently associated with ageing-dependent CI (Raihan et al., 2020), *Nrxn2*, whose mutations have been related to autism (Südhof, 2008), and in which the skipping of the AS4 exon is selectively regulated by SLM2 (Ehrmann et al., 2013, 2016), and *Arhgef9*, in which skipping of exon 11a is under the regulation of Sam68 (Farini et al., 2020) and whose mutations are associated with intellectual disability and epilepsy (Hines et al., 2022; Yang et al., 2022). Interestingly, we observed that only inclusion of *Arhgef9* exon 11a was significantly increased in the hippocampus of EAE mice (Student’s *t*-test *p* = 0.027; Figures 3A,a,b), whereas no changes in the splicing pattern of *Nrxn2* and *Srsf11* were detected (Figures 3A,a,c,d).

Since we found that EAE particularly affected the CA3 subregion of the hippocampus, we set out to analyze splicing changes separately in the main hippocampal subfields. To this end, we isolated by LMD the pyramidal layer of CA1 and CA3 and the granular layer of DG (Figure 3B). Region-specific analysis of the *Arhgef9* splicing pattern indicated that inclusion of *Arhgef9* exon11a was selectively increased in the CA3 and DG subfields of EAE mice, whereas no significant changes were observed in the CA1 subfield (Figures 3C,D). Since Sam68 promotes skipping of exon 11a (Farini et al., 2020), we then asked whether the increased inclusion of this exon was associated with modulation of *Sam68* expression. Indeed, qPCR analysis revealed that *Sam68* expression was significantly reduced only in the EAE CA3 and DG subfields, but not in the CA1 (Figure 3E). These results suggest that EAE causes region-specific changes in the splicing pattern of a synaptic gene through modulation of the expression of the splicing factor Sam68.

## 7. Discussion

In line with previous studies (Mattner et al., 2013; Coda et al., 2021), we found that PLP_139–151_-induced EAE causes marked inflammatory changes in the hippocampus of SJL/J mice at the early acute phase of the disease. Inflammation was associated with activation of microglia and increased expression of IL1β, TNFα, IL6, and IL17. These proinflammatory cytokines are known to play a crucial role in the inflammatory processes that characterize both MS and EAE (Wang et al., 2017). Moreover, they have been also shown to contribute to neuroinflammation-mediated synaptic dysfunction and the related CI that accompany the disease (Rossi et al., 2014a,b; Di Filippo et al., 2021). Our data suggest that, along with the CNS-disseminated distinctive lesions in both MS and EAE, the inflammatory process varies in intensity and effects from area to area. This phenomenon may rely on both anatomical and pathophysiological features. An example is represented by the hippocampus, where we observed a prevalent activation of microglia in the CA3 and, to a lesser extent, the DG subfields. These findings indicate a selective vulnerability of these regions to the inflammatory events related to EAE. This selectivity may rely on the specific groups of neurons with proper functional properties that are present in each subfield of the hippocampus (Oliva et al., 2016; Ayhan et al., 2021). In this regard, gene expression profiling studies revealed extensive differences among the transcriptional signatures of neurons in the main hippocampal subfields, as well as along the dorso-ventral axis of this structure (Cembrowski et al., 2016). This diversity led to the hypothesis that different regions of the hippocampus could be preferentially targeted by different disorders, including Alzheimer’s diseases (AD), schizophrenia, as well as during physiological aging (Small et al., 2011). Similarly, a regional distribution of neuroinflammation has been reported in the hippocampus of both MS patients and MOG-induced EAE mice (Planche et al., 2018). We hypothesize that the selective vulnerability of the CA3 and DG subfields could be facilitated by their particular anatomical location. Indeed, the two regions are closer than the CA1 subfield to liquoral spaces, through which cytokines and immune cells preferentially penetrate into the hippocampus of EAE mice (Mapunda et al., 2022).

The selective involvement of the CA3 subfield was also highlighted by the local increase in the size of PV + interneuron subpopulation. In the hippocampus, PV + cells in the pyramidal layer are the major source of perisomatic inhibition onto pyramidal cells, which is crucial for the generation of hippocampal network oscillations involved in spatial processing, learning and memory (Hu et al., 2014). Interestingly, an enhanced expression of PV was found in several experimental models of neurologic and psychiatric diseases associated with CI. For instance, the increased expression of PV in perisomatic boutons around pyramidal cells in CA1 and CA3 has been related to an impaired crosstalk between these hippocampal regions in a transgenic mouse model of AD (Hollnagel et al., 2019). Enhanced PV expression has also been detected in other models of neuroinflammation, such as lipopolysaccharide-induced inflammation (Ji et al., 2020). Interestingly, increased PV expression in this mouse model was reported to exert a causative role in cognitive and behavioral disturbances triggered by neuroinflammation (Ji et al., 2020). Our results now suggest that EAE induces local alterations in hippocampal GABAergic circuitry, which could contribute to impair the functional properties of this area of the brain. Indeed, as demonstrated in our previous study (Marchese et al., 2021), EAE mice show impaired performances in the novel object recognition task, which is frequently used to explore, among the others, also hippocampal functions (Chao et al., 2022). Notably, however, a discrepancy exists between our findings in PLP_139-151_-induced EAE in SJL/J mice and those found in the hippocampi of MOG-induced EAE-mice, in which a selective reduction of PV+ interneurons was found in the CA1 subfield (Nisticò et al., 2013). Differences in strains (SJL/J mice vs. C57Bl/6 mice) and/or in the clinical phenotype of disease induced by PLP_139-151_ [relapsing–remitting (RR)–EAE] with respect to the MOG_35-55_-induced model (chronic EAE) could account for the reported differences.

The regional selectivity of RR-EAE-induced hippocampal lesions was associated with a specific neuronal downregulation of *Sam68*, selectively in the CA3 and DG regions. Sam68 is a multifunctional RNA binding protein involved in mRNA transport (Klein et al., 2013), translation (Grange et al., 2009; Paronetto et al., 2009), and splicing (Matter et al., 2002; Paronetto et al., 2007; Chawla et al., 2009; Iijima et al., 2011; Farini et al., 2020). In neuronal cells it plays many relevant roles, by acting as a key regulator of activity dependent AS of synaptic proteins (Iijima et al., 2011; Farini et al., 2020) and as modulator of synaptic plasticity, through the regulation of the expression of *Arc* mRNA and metabotropic glutamate receptor-dependent LTD exclusively at distal CA1 synapses (Klein et al., 2019). Therefore, the reduced expression of *Sam68* might considerably contribute to the EAE-induced hippocampal impairment. This result is corroborated by previous studies reporting that its absence in hippocampal neurons is related with decreased spine density and altered synaptic structure (Klein et al., 2013). Consistent with the regionalized downregulation of *Sam68*, we found a specific modulation of the splicing pattern of its target *Arhgef9*, which encodes for collybistin. This protein interacts with the scaffold protein gephyrin at the level of the postsynaptic compartment of GABAergic synapses (Soykan et al., 2014). This complex plays a prominent role in the functional efficacy of the assembly of the postsynaptic neurotransmitter receptor apparatus in inhibitory synapses (Soykan et al., 2014). Interestingly, this observation fits with the regionalized changes in inflammatory features and circuitry changes here reported and suggests the possible involvement of the GABAergic system, at least during the early phases of the disease.

The functional implications of increased inclusion of exon 11a in *Arhgef9* are not known. It has been suggested that inclusion of this exon introduces a stop codon in the mRNA and yields a shorter protein isoform with a different carboxyl terminal domain (Harvey et al., 2004), possibly resulting in the assembly of a different network of proteins in the post-synaptic compartment (Farini et al., 2020). Nevertheless, it remains to be tested whether the observed changes in expression and splicing at the transcript levels, reported herein, directly translate into changes of Sam68 and Arhgef9 isoforms at the protein level. Although further studies are needed to clarify this issue, our results reinforce the hypothesis that the dysregulation of synaptic splice variants may underlie defects in specification of synapses on hippocampal neurons, thus contributing to microcircuit changes inducing hippocampal dysfunction and CI during EAE. Moreover, our findings underlie the selective vulnerability of specific CNS areas/subregions, an aspect of great relevance for MS patients, shedding a light on potential new targets involved in the progressive CI, as well as in the management of disease progression.

## Data availability statement

The raw data supporting the conclusions of this article will be made available by the authors, without undue reservation.

## Ethics statement

The animal study was reviewed and approved by Ethics Committee of animal welfare organization (OPBA) of the “Università Cattolica del Sacro Cuore” of Rome and by the Italian Ministry of Health (authorization number 321/2017-PR, protocol number 1F295.34/04-11-2016, date of approval April 12, 2017).

## Author contributions

AA, GDS, LRV, MT, and DB conducted experiments. AA, GDS, VC, and MCG performed data analysis. AA, MCG, CS, and GDS wrote or contributed to writing of the manuscript. MCG, CS, FR, and GDS reviewed and edited the manuscript. MCG and CS supervised the study. MCG, CS, and FR provided resources and funding. All authors contributed to the article and approved the submitted version.

## Funding

This research was supported by Fondazione Italiana Sclerosi Multipla to CS (FISM 2017/R/24) and Ministero della Salute - Ricerca Corrente 2022 to IRCCS Fondazione Policlinico A. Gemelli. Università Cattolica del Sacro Cuore contributed to the funding of the research work (D1. Line 2020, 2021, 2022 to CS; D1. Line 2019 to MCG; D1. Line 2019 to FR).

## Conflict of interest

The authors declare that the research was conducted in the absence of any commercial or financial relationships that could be construed as a potential conflict of interest.

## Publisher’s note

All claims expressed in this article are solely those of the authors and do not necessarily represent those of their affiliated organizations, or those of the publisher, the editors and the reviewers. Any product that may be evaluated in this article, or claim that may be made by its manufacturer, is not guaranteed or endorsed by the publisher.

## Supplementary material

SUPPLEMENTARY FIGURE S1 **(A)** Graph shows values of clinical score and symptoms (CSS) in EAE mice, evaluated as described in Materials and Methods. Data report the average clinical score and standard deviation for each group (*n*: CTRL = 5; EAE = 8). **(B)** Representative micrographs of Nissl-stained hippocampal coronal sections from CTRL and EAE mice. Perivascular and subpial infiltrates in EAE animals are indicated by arrows. Scale bar = 120 μm. **(C,D)** Graphs represent semiquantitative evaluation of the percentage of area damaged by infiltrates **(C)** and number of infiltrates/mm^2^ in different regions of EAE mice forebrain **(D),** as described in Material and methods. All values are given as means ± SD.
