# Supplementary figures and images for: Regionally restricted modulation of Sam68 expression and Arhgef9 alternative splicing in the hippocampus of a murine model of multiple sclerosis
Source: Front Mol Neurosci. 2023 Jan 12;15:1073627. doi: 10.3389/fnmol.2022.1073627 (PMC9878567; doi:10.3389/fnmol.2022.1073627)

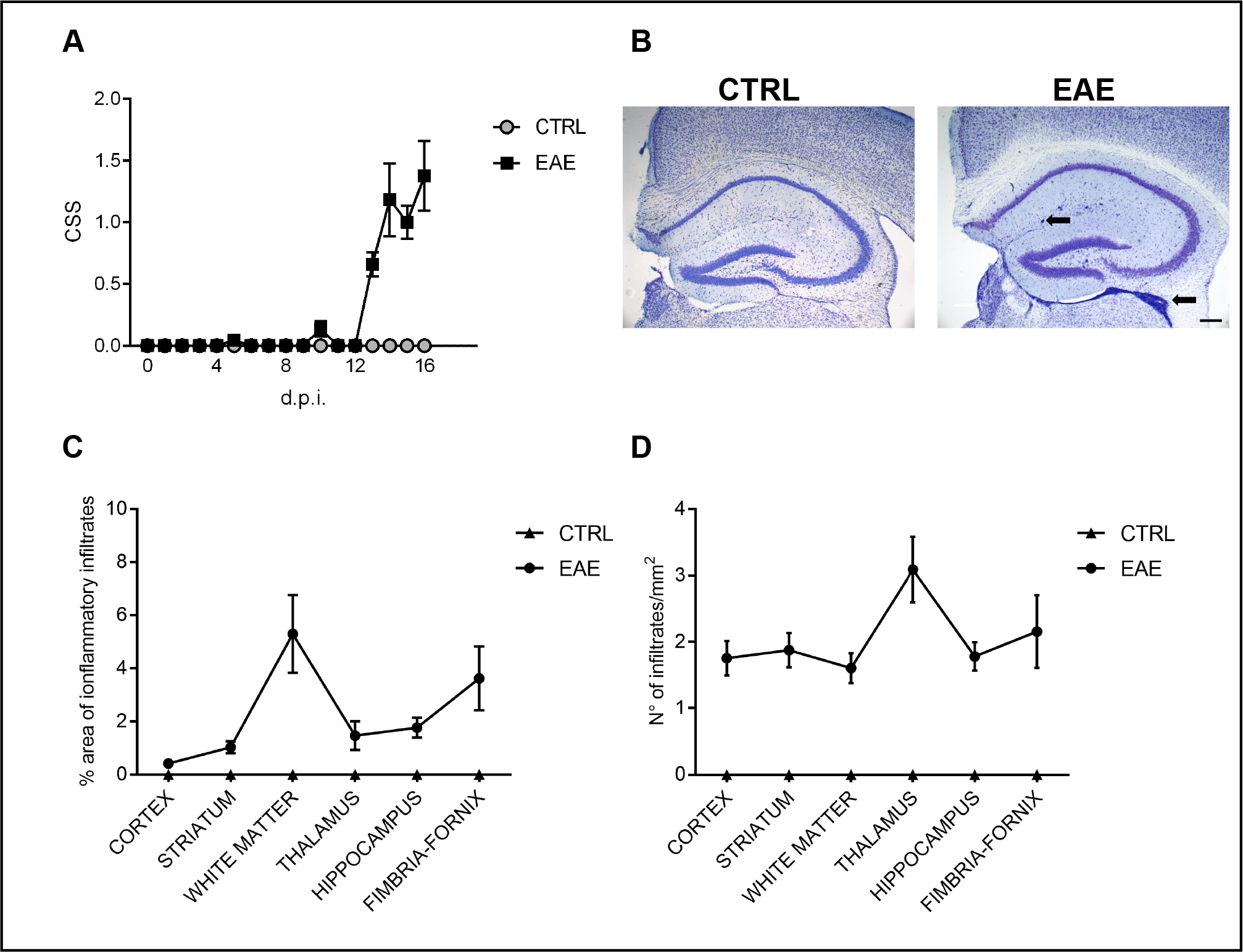

Supplement: SUPPLEMENTARY FIGURE S1 — (A) Graph shows values of clinical score and symptoms (CSS) in EAE mice, evaluated as described in Materials and Methods. Data report the average clinical score and standard deviation for each group (n: CTRL = 5; EAE = 8). (B) Representative micrographs of Nissl-stained hippocampal coronal sections from CTRL and EAE mice. Perivascular and subpial infiltrates in EAE animals are indicated by arrows. Scale bar = 120 μm. (C,D) Graphs represent semiquantitative evaluation of the percentage of area damaged by infiltrates (C) and number of infiltrates/mm2 in different regions of EAE mice forebrain (D), as described in Material and methods. All values are given as means ± SD. [file Image_1.TIF]
