# Supplementary material for: Regionally restricted modulation of Sam68 expression and Arhgef9 alternative splicing in the hippocampus of a murine model of multiple sclerosis
Source: Front Mol Neurosci. 2023 Jan 12;15:1073627. doi: 10.3389/fnmol.2022.1073627 (PMC9878567; doi:10.3389/fnmol.2022.1073627)
Supplement: Supplementary file 2 [file Table_1.docx]

| NAME | FORWARD SEQUENCE | REVERSE SEQUENCE |
| --- | --- | --- |
| Arhgef9 | 5’-CAGGCTGCAATGACTGTGAGA-3’ | 5’-ACACTTGAGACTGAGCGATGC-3’ |
| Bdnf | 5’-GGCTGACACTTTGAGCACGCT-3’ | 5’-CTCCAAAGGCACTTGACTGCTG-3’ |
| Il17 | 5’- TCAGGGTCTTCATTGCGGTG-3’ | 5’-TCTTTAACTCCCTTGGCGCA-3’ |
| Il1β | 5’-TGCCACCTTTTGACAGTGATG-3’ | 5’-ATGTGCTGCTGCGAGATTTG -3’ |
| Il6 | 5′-ACACATGTTCTCTGGGAAATC-3′ | 5’-AAGTGCATCATCGTTGTTCATACA-3’ |
| L34 | 5’-GGTGCTCAGAGGCACTCAGGATG-3’ | 5’-GTGCTTTCCCAACCTTCTTGGTGT-3’ |
| Nrxn2 | 5’- GTGCGCTTTACTCGAAGTGGTG -3’ | 5’- CCCATTGTAGTAGAGGCCGGAC -3’ |
| Pva | 5’-GCTTCTCCTCAGATGCCAGAG-3’ | 5’-TCAACCCCAATCTTGCCGTC-3’ |
| Sam68 | 5’- GACCCCCGTGTGAAGCTTAT -3’ | 5’- AGAGGGTTCAGGTACTCCG -3’ |
| Srsf11 | 5’- AGACGCTCTCGGAGTGCAAG- 3’ | 5’- CTTGTTGATCGTTCCCTTTC-3’ |
| Vgat | 5’- GGGCTGGAACGTGACAAATG-3’ | 5’- ACTGCGGCGAAGATGATGAG-3’ |

**Supplementary Table1**

**Primer sequence**
