# Supplementary material for: Regionally restricted modulation of Sam68 expression and Arhgef9 alternative splicing in the hippocampus of a murine model of multiple sclerosis
Source: Front Mol Neurosci. 2023 Jan 12;15:1073627. doi: 10.3389/fnmol.2022.1073627 (PMC9878567; doi:10.3389/fnmol.2022.1073627)
Supplement: Supplementary file 3 [file Data_Sheet_1.docx]

**Supplementary material**

**Materials and methods**

**Immunization and EAE induction**

Clinical score and symptoms scale: 0, no clinical score; 1, loss of tail tone; 2, weak hind leg paresis; 3, posterior leg paresis; 4, complete paraplegia; and 5, death. Intermediate values were used for incomplete symptoms (Penitente et al., 2008).

All experimental procedures were approved by the Italian Ministry of Health (authorization number 321/2017-PR, protocol number 1F295.34/04-11-2016, date of approval April 12^th^, 2017).

**Neuropathological evaluation**

Semi-quantitative neuropathological evaluation was performed in Nissl-stained coronal brain slices, and the following brain areas were examined: cerebral cortex, striatum, thalamus, hippocampus, white matter tracts (corpus callosum, internal capsule, fimbria). We analyzed the number of infiltrates/$\mathrm{mm}^{2}$and the percentage of area infiltrated by mononuclear cells in one or 2 adjacent sections corresponding to Figures 19-20, 25-27, 35-36, 43-45 of the atlas of Paxinos and Franklin (Paxinos and Franklin, 2013). Images were captured at the magnification of 5x using the Photomicroscope Zeiss Axiophot equipped with video camera AxioCam MRc and image analysis software ZEN 2.3, and then analysed with the Java ImageJ image processing and analysis program (*https://imagej.nih.gov/ij/, 1997-2018, NIH*). The numbers of infiltrates/$\mathrm{mm}^{2}$ or values of percentage of area infiltrated by mononuclear cells from each region/each animal were averaged and, when appropriate, used for statistical analysis (Marchese et al., 2021).

**Unbiased stereology**

The optical fractionator stereological design was used to obtain unbiased counts of total numbers of PV-positive (+) interneurons in the CA1, CA3 and DG hippocampal subregions using the Stereo Investigator system (Stereo Investigator software, Version 9, MicroBrightField Europe, Germany), essentially as previously described (Corvino et al., 2015; Marchese et al., 2018).

Briefly, a stack of MAC 6000 controller modules (MBF Bioscience, Williston VT, United States) was configured to interface with a Nikon Eclipse 80i microscope with a motorized stage and a digital color camera (MBF Bioscience q imaging) with a Pentium II PC workstation. PV+ cells were counted in 7 immuno-stained serial coronal sections; a three-dimensional optical dissector counting probe (x, y, z dimension of 150 *µ*m x 150 *µ*m x10 *µ*m, respectively) was applied to a systematic random sample of sites in the region of interest (magnification: 40X).

**Confocal microscope quantitative analysis of double-stained cells**

Iba1/CD68 double-stained cells were quantified in the CA1, CA3 and DG hippocampal subfields of EAE and CTRL mice using z-scan confocal microscopy at 40X magnification, as previously described (Corvino et al., 2015; Marchese et al., 2018). The number of double-labeled cells was counted manually (Corvino et al., 2015; Marchese et al., 2018). Cell counts were corrected for the effects of counting fragments of cells whose centers are not located within the thickness of the section, according to Abercrombie’s correction, as previously described (Corvino et al., 2015; Geloso et al., 1998; Marchese et al., 2018)
